# Comparing methods of determining *Legionella spp.* in complex water matrices

**DOI:** 10.1186/s12866-015-0423-7

**Published:** 2015-04-29

**Authors:** Álvaro Díaz-Flores, Juan Carlos Montero, Francisco Javier Castro, Eva María Alejandres, Carmen Bayón, Inmaculada Solís, Roberto Fernández-Lafuente, Guillermo Rodríguez

**Affiliations:** Departamento de Microbiología General III, Facultad de Ciencias Biológicas, Universidad Complutense de Madrid, Campus Moncloa, 28040 Madrid, Spain; Instituto de Ciencias de la Salud Ctra, de Extremadura Km. 114, 45600 Talavera de la Reina, Spain; Laboratorio Regional de Salud Pública Consejería de Sanidad y Consumo/Comunidad de Madrid, C/ Sierra del Alquife N 8, 2 Planta, 28053 Madrid, Spain; Iproma, S.L, Cno.de la Raya 46, 12005 Castellón, Spain; Departamento de Biocatálisis, Instituto de Catálisis y Petroleoquímica, Consejo Superior de Investigaciones Científicas, Campus UAM-CSIC, 28049 Cantoblanco Madrid, Spain; Biótica, Bioquímica Analítica, S.L, Science and Technology Park of Jaume I University, Campus RiuSec - Espaitec 2, planta baja, E12071 Castellón de la Plana, Spain

**Keywords:** *Legionella*, Detection, Environmental samples, Magnetic particles

## Abstract

**Background:**

*Legionella* testing conducted at environmental laboratories plays an essential role in assessing the risk of disease transmission associated with water systems. However, drawbacks of culture-based methodology used for *Legionella* enumeration can have great impact on the results and interpretation which together can lead to underestimation of the actual risk. Up to 20% of the samples analysed by these laboratories produced inconclusive results, making effective risk management impossible. Overgrowth of competing microbiota was reported as an important factor for culture failure. For quantitative polymerase chain reaction (qPCR), the interpretation of the results from the environmental samples still remains a challenge. Inhibitors may cause up to 10% of inconclusive results. This study compared a quantitative method based on immunomagnetic separation (IMS method) with culture and qPCR, as a new approach to routine monitoring of *Legionella*.

**Results:**

First, pilot studies evaluated the recovery and detectability of *Legionella spp* using an IMS method, in the presence of microbiota and biocides. The IMS method results were not affected by microbiota while culture counts were significantly reduced (1.4 log) or negative in the same samples. Damage by biocides of viable *Legionella* was detected by the IMS method. Secondly, a total of 65 water samples were assayed by all three techniques (culture, qPCR and the IMS method). Of these, 27 (41.5%) were recorded as positive by at least one test. *Legionella spp* was detected by culture in 7 (25.9%) of the 27 samples. Eighteen (66.7%) of the 27 samples were positive by the IMS method, thirteen of them reporting counts below 10^3^ colony forming units per liter (CFU l^−1^), six presented interfering microbiota and three presented PCR inhibition. Of the 65 water samples, 24 presented interfering microbiota by culture and 8 presented partial or complete inhibition of the PCR reaction. So the rate of inconclusive results of culture and PCR was 36.9 and 12.3%, respectively, without any inconclusive results reported for the IMS method.

**Conclusion:**

The IMS method generally improved the recovery and detectability of *Legionella* in environmental matrices, suggesting the possibility to use IMS method as valuable indicator of risk. Thus, this method may significantly improve our knowledge about the exposure risk to these bacteria, allowing us to implement evidence-based monitoring and disinfection strategies.

## Background

Legionellosis refers to a range of clinical syndromes as a consequence of *Legionella* infection. *Legionella spp* is the causative agent of legionellosis and has been identified as an increasing public health concern since 1976. To date, this opportunistic pathogen has been responsible for the death of thousands of people worldwide. Since its identification as a human pathogen, at least 24 out of more than 50 recognized species of *Legionella* have been associated with human diseases [1-4].

*Legionella* bacteria are omnipresent in both natural and anthropogenic aquatic environments [5,6]. Natural environments do not support extensive *Legionella* growth but anthropogenic systems can promote its proliferation to high concentrations. Abatement of *Legionella* bacteria appears to be difficult and environmental eradication is not possible. *Legionella* can transform itself into viable but non culturable (VBNC) and persistent forms, as well as grow on necrotrophic substrate and survive in protozoa and biofilm, compromising the efficiency of control strategies based on chemical, mechanical and physical disinfection systems [7-10]. In this context, prevention of legionnaires’ disease requires a proactive evidence-based approach comprising both the accurate identification and assessment of the threat of *Legionella* bacteria in risk facilities and the appropriate application of supplemental disinfection treatments [11]. As the population ages, the health impact on ‘at risk’ groups of legionellosis is likely to continue to increase in the absence of more effective prevention measures and/or improved implementation of prevention measures [12].

The World Health Organization published documents addressing *Legionella* prevention in man-made water systems. Regular checking of the *Legionella* level has been recommended to examine trends in *Legionella* concentration and to verify and validate water safety plans [13]. Research on environmental monitoring may be beneficial to evaluate methods to quantify *Legionella* levels in water systems as well as to define more clearly the role of routine environmental monitoring as a guide to remediation. The first step in the management of environmental prevention is timely detection of target organisms in the potential sources of infection [14].

Official methods for *Legionella* detection are based on the growth of the microorganism in selective media [15,16]. Long assay time, low sensitivity, loss of viability after collection or sample treatment, presence of interfering microbiota and the inability to detect VBNC state, are well documented limitations of this growth-based technique [17-24]. Quantitative polymerase chain reaction (qPCR) has been proposed as method for monitoring *Legionella* in environmental systems [25,26], but the interpretation of qPCR results from environmental samples remains difficult [27,28]. The main problem of qPCR is that it enumerates DNA of both live and dead cells leading to an overestimation of the actual health risk [29,30]. The feasibility and the added value of techniques that differentiate DNA from live and dead bacteria (ethidium/propidiummonoazide staining) or that detect *Legionella* RNA need to be further evaluated on water samples that may be complex matrices [31].

Moreover, PCR inhibiting compounds present in environmental samples may potentially lead to inaccurate target quantification or false-negative results. Many water sources are known to contain PCR inhibitors which may become concentrated on the filters and carryover to the final DNA extraction [32]. Such inhibitors adversely affect PCR reaction efficiency [33]. PCR inhibitors may consist of divalent cations, minerals, or other debris that may antagonize the polymerase and decrease amplification efficiency [34].

The immobilization of antibodies onto the surface of magnetic beads to obtain immunomagnetic beads (IMB) has promoted the development of immunomagnetic separation (IMS). Thereby, IMS provides a simple but powerful method for specific capture, recovery and concentration of the desired microorganism from heterogeneous bacterial suspension [35]. Immunomagnetic separation has also been combined with other detection methods for *Legionella* such as culture [36], PCR [37] or flow cytometry [38].

In this study, different laboratories used a test based on IMS by anti-*Legionella spp.* immuno-modified magnetic beads, coupled to enzyme-linked colorimetric detection for the rapid detection of *Legionella spp.* cells in water samples [39,40]. Antibodies were bound (through its Fc region) to the fairly inert bead surface. The immuno-modified beads were mixed with a sample to allow the antibodies to bind to the cell surface antigens in certain physic-chemical conditions. In these conditions, stability of this capture depends on the number of antibody-antigen interactions which is related to the cell surface integrity. So this test was expected to be able to detect the loss of viability when a cell envelope is damaged.

This study aims to compare specificity, sensitivity and accuracy of detection and quantification of the three techniques and to evaluate comparatively their suitability as a method for detection and enumeration of *Legionella* at risk facilities.

## Results

### Comparative trial with interfering microbiota

The influence of background organisms on the determination of *Legionella* in water was investigated (Table 1). Two different microbial mixtures (Microbiota I, Microbiota II) were prepared and tested negative for the IMS method. These microbial mixtures consisted mainly of background organisms usually present at water from cooling tower. The background organisms were added to water inoculated with viable *Legionella pneumophila* serogroup 1. Previously the water matrix was tested negative for both methods. The results suggested that the presence of a large number of background bacteria in the water sample could reduce the growth of *Legionella pneumophila*. Inhibition was more pronounced with one of the mixtures (Microbiota II) containing *Pseudomonas aeruginosa and Ascomycetes*. This preliminary study suggested the importance of the background organisms in water for inhibition of growth of *Legionella organisms*. No significant effect was observed on the signal of the IMS method. Nevertheless this effect may be difficult to demonstrate with a sample size as small as three independent experiments for each group so further research is needed.

**Table 1:** **Effect of microbiota on immunomagnetic method and the standard culture method**

**Type of sample**	**Sample no.**	**Results, CFU l** ^**−1**^ **(Log**_**10**_ **)**	
		**Culture method**	**IMS method**
Water Matrix	1	ND	ND
	2	ND	ND
	3	ND	ND
*L.pneumophila*	4	3.3	3.4
	5	3.4	3.5
	6	3.1	3.7
Microbiota I	7	3.0	3.3
	8	3.5	3.7
	9	ND	3.7
Microbiota II	10	ND	2.5
	11	ND	3.1
	12	ND	3.5

### Sensing effect of biocides by IMS method

The loss or severe reduction in the IMS method signal after exposure of approximately 1.0 × 10^3^ colony forming units per milliliter (CFU ml^−1^) to 3 ppm of hypochlorite or 2,2-dibromo-3-nitrilopropionamide (DBNPA) 20% can be seen in Table 2. Reduction in IMS method signal was less evident after exposure of *L. pneumophila* to 100 ppm of Mefacide than other biocides. Mefacide is a biocide based on isothiazolinone requiring to be transported inside the cell.

**Table 2:** **Effect of various biocide treatments on the IMS method test signal**

**Biocide**	**Contact time (min)**	**Reduction in signal for IMS method (%)**
Hyplochlorite, 3 ppm	60	99
DBNPA 20%	60	90
Mefacide, 100 ppm	60	5

### Comparative trial among culture, qPCR and IMS method

**Figure 1:**
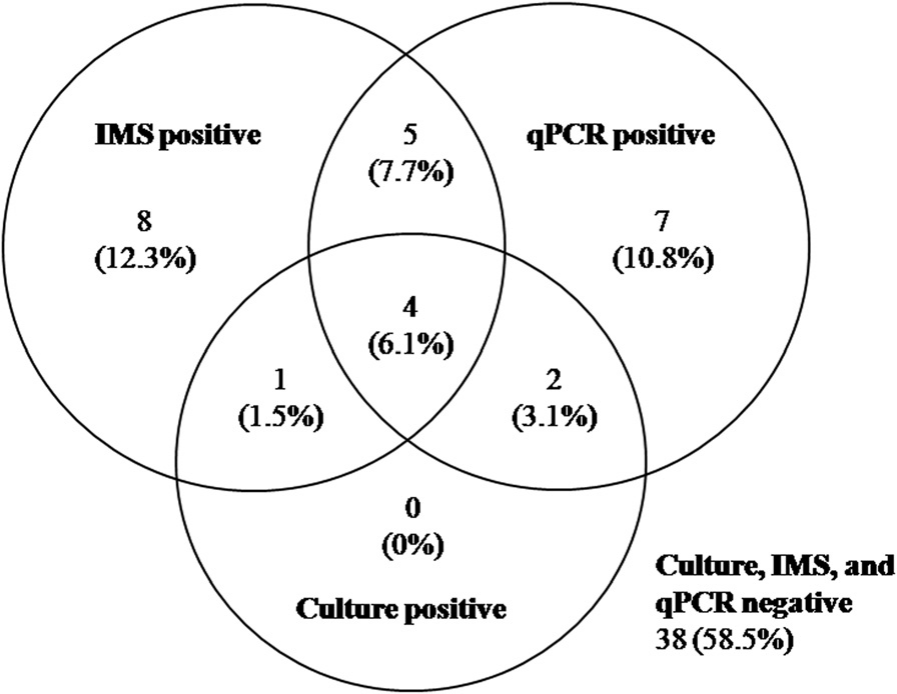
Agreement among IMS method, qPCR, and culture results.

A total of 65 water samples were assayed by all three techniques (culture, qPCR and IMS method). Of these, 27 (41.5%) were recorded as positive by at least one test. *Legionella spp* was detected by culture in 7 (25.9%) of these 27 samples. Eighteen (66.7%) of the 27 samples were positive by IMS method, and eighteen (66.7%) were PCR positive. The proportion of samples positive by PCR or IMS method was significantly greater than those positive by culture. Of the 18 IMS method-positive samples, 4 were also positive by PCR and culture, 9 were positive by PCR, and 5 were positive by culture, leaving 8 (12.3%) discrepant samples positive by the IMS method alone. One of the 5 culture-positive samples gave complete inhibition for PCR. Presentation of the results from the three tests is shown in Figure 1.

Of the eighteen samples positive by the IMS method, thirteen reported counts below 10^3^ colony forming units per liter (CFU l^−1^), six reported containing interfering microbiota and three reported PCR inhibition. Of the eight samples positive for the IMS method alone, one sample showing quantitative result >10^4^ equivalent CFU l^−1^ by the IMS method was undetected (partial inhibition) by PCR and contained interfering microbiota. The other seven samples (87.5%) showed quantitative results < 6 × 10^2^ CFU l^−1^. Of these seven samples, one showed inhibitors for PCR and other one showed competing background bacteria.

Of these 65 water samples, 24 were reported as containing interfering microbiota by culture and 8 were reported as containing partial (4) or complete (4) inhibitors of PCR (Table 3). So the rate of inconclusive results of culture and PCR were 36.9 and 12.3%, respectively. Interfering microbiota was diverse and colonies with different morphology and color were isolated (silver, yellow and brown colonies). Compared to that of culture, the sensitivities of the IMS method and qPCR were 71.4 and 85.7%, respectively. The performance characteristics of culture, IMS method and qPCR were recalculated considering the inclusion of samples positive by both PCR and IMS. Then, sensitivities of culture, IMS and PCR were 58.3, 83.3, and 91.7%, respectively.

**Table 3:** **Distribution of samples for the presence or absence of microbiota and PCR inhibition**

		**Culture number**		**qPCR number**	
**Matrix**		**Microbiota**	**No microbiota**	**Inhibition**	**No inhibition**
Cooling towers					
	Detected	3	0	0	6
	Undetected	6	7	1	13
Hot/cold sanitary					
	Detected	2	2	0	12
	Undetected	13	32	7*	26
Total		24	41	8	57

*Four out of seven presented partial inhibition (only one of two replicates was inhibited).

Data were also examined from the point of view of decisions dependent on levels of action and alert as defined in European Guidelines. The alert and action levels for the IMS method were the same as for culture, because high correspondence between the two methods exists. The two tests would have resulted in identical responses for 93.8% of comparisons (Table 4). In just one case culture indicated no action is required while the IMS method indicated emergency immediate action. This case corresponded to a sample presenting PCR inhibitors, so it was also not detected by PCR.

**Table 4:** **Comparison of action**/**alert levels using immunomagnetic separation based method (IMS) and culture for** ***Legionella spp***

			**IMS method no.**			
			**Action**	**Alert**	**Satisfactory**	**Total**
***Legionellaspp***			**≥10** ^**4**^ **CFU l** ^**−1**^	**≥10** ^**3**^ **CFU l** ^**−1**^	**<10** ^**3**^ **CFU l** ^**−1**^	
Culture no.	Action	≥10^4^ CFU l^−1^	0	0	0	0
	Alert	≥10^3^ CFU l^−1^	0	2	1	3
	Satisfactory	<10^3^ CFU l^−1^	1	2	59	62
Total			1	4	60	65

Recently, qPCR action and alert levels were proposed [25]. PCR selected levels were those used for the culture, adjusted by corresponding mean log difference between quantitative PCR results reported in genomic units per liter (GU l^−1^) and culture results (CFU l^−1^) in the conducted study. Reported mean log difference for *Legionella* spp. was 1.05 log and 2.03 log for hot and cold water and cooling towers, respectively. The mean log differences found in this study were much lower than that reported by Lee et al. [25], −0.1 log and 0.64 log for cooling towers and hot and cold sanitary water, respectively. As the sample size of cited report was larger than those applied in this study, the levels established in that report for *Legionella* spp. were assumed.

Considering *Legionella* spp target, for 89.1% of comparisons, use of the two tests (qPCR and IMS method) would have resulted in identical responses (Table 5). Additional 18 water samples analysed by both techniques were also included.

**Table 5:** **Comparison of action/alert levels using quantitative PCR (qPCR) and immunomagnetic separation based method (IMS) for** ***Legionella spp***

				**IMS method no.**			
***Legionellaspp***				**Action**	**Alert**	**Satisfactory**	**Total**
		**Hot/cold sanitary**	**Cooling towers**	**≥10** ^**4**^ **CFU l** ^**−1**^	**≥10** ^**3**^ **CFU l** ^**−1**^	**<10** ^**3**^ **CFU l** ^**−1**^	
	Action	≥10^5^ GU l^−1^	≥10^6^ GU l^−1^	10	2	0	12
qPCR no.	Alert	≥10^4^ GU l^−1^	≥10^5^ GU l^−1^	0	2	1	3
	Satisfactory	<10^4^ GU l^−1^	< 10^5^ GU l^−1^	1	5	62	68
	Total			11	9	63	83

Both IMS method (Table 4) and qPCR (Table 6), for 93.8% of comparisons, would have resulted in identical responses that those derived from culture results.

**Table 6:** **Comparison of action/alert levels using quantitative PCR (qPCR) and culture for** ***Legionella spp***

			**qPCR no.**			
			**Action**	**Alert**	**Satisfactory**	**Total**
***Legionellaspp***		**Hot/cold sanitary**	**≥10** ^**5**^ **GU l** ^**−1**^	**≥10** ^**4**^ **GU l** ^**−1**^	**< 10** ^**4**^ **GU l** ^**−1**^	
		**Cooling towers**	**≥10** ^**6**^ **GU l** ^**−1**^	**≥10** ^**5**^ **GU l** ^**−1**^	**< 10** ^**5**^ **GU l** ^**−1**^	
	Action	≥10^4^ CFU l^−1^	0	0	0	0
Culture no.	Alert	≥10^3^ CFU l^−1^	0	0	3	3
	Satisfactory	<10^3^ CFU l^−1^	0	1	61	62
	Total		0	1	64	65

## Discussion

Routine testing for *Legionella* is required by most regulatory bodies despite the uncertainties of current quantification methods. Of the existing methods, culture is considered the “gold standard” and qPCR has been considered a very promising tool. Culture enumeration can underestimate the risk of *Legionella* due to, among others issues, inability to count viable but non-culturable (VBNC) organisms, slow growth rate of *Legionella* in a plate, overgrowing of accompanying organisms, presence of vesicles containing *Legionella* expelled from protozoa, or loss of cultivability during sample holding time prior culturing. For some samples containing PCR inhibitors, high quantification limits do not allow the quantification of the target by this technique in complex waters [26]. There is a significant discrepancy between qPCR results and culture results for *Legionella* in water samples, because positivity rates for qPCR are usually greater than those obtained by culture [27]. Both accompanying organisms and inhibitors may cause a rate of inconclusive results greater than 10–20% by these two techniques.

The purpose of this study was to compare three different techniques for the routine monitoring of *Legionella spp* in waters: culture, qPCR, and an IMS method whose extensive validation has been reported in the literature [39,40]. Quantification of legionellosis risk requires enumeration of *Legionella* from an environmental source. High levels of *Legionella spp* in water (10^4^-10^10^ CFU l^−1^) are considered a risk of infection [41-44]. Underestimating the risk of *Legionella* may have serious public health consequences and overestimating the risk may result in significant economic costs [45]. In this study, laboratories were also concerned by the speed of the analysis because culture method can take up to 14 days to obtain a result, and the results are often variable with poor recovery, whereas qPCR can take 1 working day. Therefore it could be difficult to draw timely conclusions on the risk using the values from water samples by culture or qPCR when microbiota or PCR-inhibitory compounds are present. Minimizing these uncertainties, in part due to effects of natural water matrices, should result in improved management protocols.

In this context, participating laboratories explored an IMS method as a new approach to detecting and quantifying *Legionella* in a pre-concentrated water sample, in just 1 hour, for the intended purpose of prevention. The rate of inconclusive results found in this study for culture (36.9%) and qPCR (12.3%) confirmed the limitations of these two techniques anticipated by other studies. The results suggest that the performance of PCR and culture techniques are more influenced than the IMS by the characteristics of the water matrix (background microorganisms, inhibitory substances). This may serve to explain why the major discrepancy of the results was observed in the more dirty samples, were also more susceptible to colonization by *Legionella*.

This study also confirmed the suitability of the IMS method test for the detection and quantification of *Legionella spp.* in water samples. The final protocol comprised sample pre-concentration by filtration and resuspension, magnetic capture using immunoactivated beads, and colorimetric enzyme-linked immunodetection in just 1 h of analysis. Immunomagnetic separation of captured microbial target allowed minimizing matrix effects providing a better recovery of the *Legionella* present in the sample. Results in this study indicate that the IMS method could provide a more reliable detection of the viable target. Both background organisms and PCR-inhibitory compounds may be removed from a sample without loss of sensitivity through dilution, simultaneously providing the concentration of the target.

Immunomagnetic separation introduced a purification step to detect target cells separated from debris or other cells, and the direct analysis on the washed complexes of *Legionella* and beads avoids the loss of cells in a detachment step. The IMS method used *Legionella*-specific polyclonal antibody-coated beads so a broader spectrum of suitable antigens on the bacterial surface can be attributed to contributing to an increase in the likelihood of detection. Thereby it seems that the IMS method is able to detect an intact whole cell target more effectively. This occurs even though some of the samples (mainly from cooling towers) presented dirtiness that made handling difficult. Thus, the IMS method reduced the likelihood of inconclusive results in *Legionella* testing. Results indicated that the IMS method could be a more reliable option, particularly in the analysis of water samples with high levels of contamination.

Results also showed that the IMS method distinguished between intact cells and cells damaged by biocides at level of cell envelope integrity. This could be explained because the IMS method introduced extensive washing of bacteria-beads complexes by selected working buffers while shaking, removing loosely bound bacteria. The number of interactions between bead surface and damaged cell surface was likely to be insufficient. In fact, biocides which are harmful to the antigens exposed at the cell envelope caused rapid loss of IMS method signal as opposed to the biocides that need to be incorporated inside the cell to work. Therefore, this IMS method can provide an indication of cell viability based on the integrity of the outer cell envelope, depending on the action mechanism of the applied biocide.

Many molecular viability markers have been proposed to distinguish between live and dead *Legionella* cells. Generally, these markers need to be incorporated inside the cell to react with nucleic acids under certain experimental conditions, so they need to pass through a very complex cell envelope. Moreover, this envelope is thickened in the infective stage of *Legionella*, so it can be regarded as a ‘complex multi-barrier system’ [46]. It should be expected that the complexity and phenotypic plasticity of the *Legionella* cell envelope would influence on the efficacy of the marker uptake. This efficacy will also depend on the intrinsic chemical characteristics of the marker, the range of experimental conditions and the composition of the natural water samples. It is therefore necessary to conduct intensive preliminary laboratory-based experiments to optimize application protocols. The IMS method is specific to outer envelope integrity. As this outer envelope presents characteristic antigenic structures, correlated with virulence properties, the strategy used in this study was based on the control of interactions between beads activated with antibodies and this outer envelope to target the capture of undamaged cells.

During this study, few positive PCR or positive IMS method results were confirmed by culture (approximately 8% in both cases). This could be explained by the frequent presence of contaminating microorganisms that interfere with *Legionella* growth (36.9%), which lead to decreased sensitivity. Moreover, *Legionella* cells that are viable but non-culturable are not detected by conventional culture.

The difference in positivity rates was due largely to false-negative culture results rather than to false-positive results by PCR or IMS method. Concerns remains over PCR false-positive results due to contamination with dead cells or free DNA. Moreover, eight samples (12.3%) showed complete or partial PCR inhibition. Neither DNA nor damaged envelope cells were detected by IMS method.

Interestingly the mean log difference between quantitative PCR result (GU/L) and IMS method result (equivalent CFU l^−1^) in natural water samples was 0.70 (SD = 1.02) based on 9 pairs of samples in which *Legionella spp.* were detected by both methods. In contrast for artificial water samples, there was 8 pairs of samples for which the mean log difference was −0.05 (SD = 0.16). Only pairs of results with readings above the quantification limit have been used. This probably reflects the fact that practically all DNA target for PCR was contained into viable cells inoculated in artificial samples, which are the target of the IMS method at the same time. However, a fraction of DNA detected by PCR in environmental samples might be free or belonging to dead or damaged cells, without sanitary risk.

In view of the difficulty to find a correlation between qPCR and culture, data analysis to derive action and alert levels has been reported. This analysis adjusted qPCR action levels to achieve a high proportion of results in the boxes indicating agreement in the actions required and to minimize any results in those corresponding to complete disagreement. This adjustment was based on the mean difference found in the study for *Legionella* spp. in cooling towers, 2.03 (SD = 1.07), and hot and cold water system, 1.05 (SD = 0.81). This criterion is dependent on the nature of the system and its treatment. The IMS method used in this study has a demonstrated correlation with culture method [40]. Therefore the IMS method uses the same action and alert levels still defined for the culture by European Guidelines while overcoming the major drawbacks of the growth-based techniques.

For three comparisons between qPCR and culture method, there was partial disagreement with culture indicating an alert response when qPCR was satisfactory as opposed to one comparison with qPCR indicating an alert response when culture was satisfactory. For one comparison between the IMS and culture methods, there was partial disagreement with culture indicating satisfactory response when IMS method indicated alert as opposed to two comparisons with culture indicating alert when IMS method indicated satisfactory response. Moreover, for one comparison there was complete disagreement with culture indicating satisfactory response when the IMS method indicated a requirement for emergency action. For this last sample, both interfering microbiota for culture and possible qPCR inhibition (dirty sample) were reported. As the detection step in the IMS method is always performed at the end of analysis when the captured target has been purified, the conditions of the final measurement are consistent and independent of the water matrix. This fact can simplify the process and renders the IMS method suitable for routine monitoring in sanitary inspection and surveillance.

## Conclusions

The methods tested in the present study might be used in laboratories for routine water analysis. Both culture and qPCR methods were more influenced by matrix effects than the IMS method. The IMS method provides a testing protocol based on immunomagnetic purification to reduce the rate of inconclusive results for the purpose of risk assessment and management legislation. In future it will be possible to derive algorithms for the use of IMS method for routine monitoring at laboratories and risk facilities. From the data collected in this study and from others, these algorithms will use the same alert and action levels that those defined by culture, with the benefit to avoid interference from background organisms or substances.

## Methods

### Participating laboratories

Three laboratories participated in this study. Of these laboratories, two were public health laboratories conducting *Legionella* testing in its routine work. The other one was a private laboratory also accredited for *Legionella* testing that are regularly testing water samples for clients maintaining facilities. All laboratories were experienced in the detection and isolation of legionellae by culture and PCR and demonstrated competence by their performance in external quality assurance schemes for *Legionella* isolation. To ensure that all laboratories were able to use the immunomagnetic method reliably, a training trial was performed at the beginning of the study. Laboratory 1 performed the standard ISO 11731 method and the IMS method on water samples containing target organism and different mixtures of interfering microbiota. Laboratory 2 performed the IMS method on water samples with and without different biocides. Laboratory 3 conducted a comparison study with all three techniques: the IMS method, qPCR, and culture.

### Trial with interfering microbiota

*Legionella pneumophila* serogroup 1 (ATCC 33152) was provided by Eurofins (France). Environmental isolates of no-*Legionellae* microorganisms usually present at environmental water samples was used to prepare two mixtures of potentially interfering microbiota. The Mixture I consisted of *Pseudomonas aeruginosa*, *Escherichia coli*, and *Streptococcus faecalis*. The Mixture II consisted of *Pseudomonas aeruginosa*, and *Ascomycetes*. A water matrix tested negative for both methods was selected. Three groups of microbial samples were prepared. The first group consisted of three independent 250 ml-portions of this matrix, each spiked with 2 × 10^4^ CFU of *Legionella pneumophila* serogroup 1. The second group consisted of three independent 250 ml-portions of this matrix, each portion spiked with 2 × 10^4^ CFU of *Legionella pneumophila* serogroup 1 and 10^8^ CFU of each microorganism belonging to the mixture I. The third group consisted of three independent 250 ml-portions of this matrix, each portion spiked with 2 × 10^4^ CFU of *Legionella pneumophila* serogroup 1, 10^8^ CFU of *Pseudomonas aeruginosa* and 5 × 10^3^ CFU of *Ascomycetes*, corresponding to mixture II. All 250 mL-portions were assayed by both culture and the IMS method.

### Trial with biocides

Three biocides were selected for this experiment. Hypochlorite (oxidizing agent) and DBNPA (not oxidizing agent) did not need to be internalized inside the cell to act on the cell, and Mefacide (based on isothiolizonona) need to be internalized because it acts on metabolism. Five independent experiments were conducted. For each independent experiment a suspension of *Legionella pneumophila* serogroup 1 was prepared and divided into four portions. One portion without biocide was considered as positive control and the other three portions were mixed each one with a 1 ppm of hypochlorite, 50 ppm of DBNPA 20% and 100 ppm of Mefacide, respectively. After 1 hour contact time, both control and samples were assayed by the IMS method.

### Comparative trial

#### Sampling

Water samples (a total of 65) were collected from both urban and rural areas of Castilla La Mancha and Madrid. Water samples included different matrices as cooling tower, sanitary water (hot/cold), nebulizer and spa matrices. Water samples of 2 L from were collected in accordance with ISO 19458:2006 into sterile containers containing sodium thiosulphate to neutralize any residual oxidizing biocides in the water. Samples were transported to the laboratory as soon as possible, and processed within 24 h of collection. Eighteen additional artificial samples were prepared by spiking *Legionella pneumophila* serogroup 1 ATCC33152 (Bioréférence, Eurofins) and were analysed by qPCR and the IMS method.

#### Filtration and resuspension of cells from water samples

Each sample was mixed well by shaking by hand then filtered through a 2.7 μm glass fiber pre-filter (Filterlab) and a 0.4 μm nylon filter (Millipore) overlapped. Pre-filtration allowed separation of bacteria from bigger particles and this was discarded after filtration. The filter was then removed from the filter holder and placed with 20 ml of the diluent L0 (Biótica) in a 100 ml sterile plastic container, vigorously vortexed for 2 minutes.

Each 20 ml concentrated sample was thoroughly mixed and then divided into three portions. One 10 ml portion was assayed by qPCR (Applied Biosystems) for *Legionella spp.* with internal process controls in order to assess inhibition or suboptimal reaction conditions. The second 1 ml portion was assayed by culture for *Legionella species* following ISO 11731. The third 9 ml portion was assayed by the IMS method (Legipid, Biótica).

All three techniques were applied to the same concentrated sample, so results were not affected by variety of filtration/resuspension step.

### Reference culture method

Culture procedure followed the ISO standard 11731–1:2004. 0.1-0.5 ml portions of concentrated sample were cultured onto the selective medium GVPC without pretreatments, at 36°C for 10 days. Presumptive colonies were cultivated on buffered charcoal yeast extract media, BCYE and BCYE-Cys, at 36°C during at least 2 days. Colonies grown on BCYE but not on BCYE-Cys were confirmed as *Legionella*. Moreover, agglutination latex test was also applied for suspicious colonies.

### Immunomagnetic method

9 ml portion of each concentrated sample was assayed by IMS method (Legipid, Biótica). Assays were conducted according to the instructions of manufacturer. The immunomagnetic procedure consisted of three steps: *Legionella*-capturing with magnetic beads activated with immobilized antibodies against *Legionella*, washing of the complexes *Legionella*-beads, *Legionella*-labeling with enzyme conjugated antibodies against *Legionella*, washing of the labeled complexes, and colorimetric reaction. The protocol from Biótica was applied. A negative control was tested in parallel for subtracting the signal of unspecific adsorption from the signal of the tested sample. The specificity of the IMS method has been evaluated [39]. The IMS method derived equivalent CFU results because there is a correspondence between IMS and culture methods [40].

### qPCR *Legionella spp* assay

10 ml portions of concentrated sample in falcon tubes were centrifugated at 2,000 rpm for 10 minutes, obtaining 350 μl of the supernatant. After addition of 50 μl of reaction buffer on each supernantant, two replicates of 10 μl were assayed.

(i) **DNA extraction.** 50 μl of lysis reagent was added on each microcentrifuge tube containing the supernatant, to facilitate the cell membrane breakage. The tubes were vortexed and then incubated at 95°C for 10 min in a thermomixer. Following incubation, tubes were left to equilibrate at room temperature for 5 minutes. Thetubes were then vortexed and centrifuged at 6,000 rpm for 2 minutes and each supernatant was transferred to an eppendorf tube. Extracted DNA from concentrated samples was added to PCR mixtures immediately or stored at −20°C a maximum of 2 days.
(ii) ** DNA amplification.** PCR tubes were placed in a model StepOne 96-well thermal cycler (Applied Biosystems). Two 20-base oligonucleotides were used as amplimers enclosing a 386-bp fragment of the 16S rRNA gene. p1.2 (59-AGGGTTGATAGGTTAAGAGC-39) was located at positions 451 to 470, and cp3.2 (59-CCAACAGCTAGTTGACATCG-39) was complementary to positions 836 to 817.The amplification reactions were performed in optical microplates using a total volume of 25 μl. Ten microliter of extracted DNA was added to each well on 15 μl of PCR mix containing thermostable Taq polymerase and specific probe for *Legionella* spp (gen 16 s, 386 pb (451–837)). All samples were amplified in duplicate, reporting the average of the two obtained results. The reaction mixtures contained 1× TaqMan universal PCR master mix (PCR buffer, deoxynucleoside triphosphates, AmpliTaq Gold polymerase, Amp Erase uracil *N*-glycosylase [UNG], MgCl_2_; Life Technologies, Madrid, Spain), 300 nM of each *Legionella*-specific primer, 250 nM TaqMan Minor Grove Binding (MGB) *Legionella*-specific probe labeled with 6-carboxy fluorescein (FAM) - excitation and emission wavelengths of 495 and 515 nm respectively- , and 250 nM TaqMan Minor Groove Binding (MGB) probe labeled with VIC - excitation and emission wavelengths of 528 and 546 nm respectively-, to detect internal control of the process (IPC).
(iii) ** Quantification.** Quantitative results were obtained by a calibration curve in the range of 10–100,000 genomic units (GU), with five levels and three replicates per level. Included in each run were three negative controls and two positive controls, and one internal positive control (IPC) for each sample. The inclusion of the IPC in each reaction avoids false negatives due to the presence of substances that inhibit PCR. The IPC signal proves that PCR reagents are working and amplifying satisfactorily. The inhibition is reported as partial if the IPC is inhibited in one of the replicates, and it is reported as complete if the IPC is inhibited in the two replicates.
